# A Case of Primary Combined Squamous Cell Carcinoma with Neuroendocrine (Atypical Carcinoid) Tumor in the Floor of the Mouth

**DOI:** 10.1155/2016/7532805

**Published:** 2016-12-27

**Authors:** Kenji Yamagata, Kazuhiro Terada, Fumihiko Uchida, Naomi Kanno, Shogo Hasegawa, Toru Yanagawa, Hiroki Bukawa

**Affiliations:** Department of Oral and Maxillofacial Surgery, Institute of Clinical Medicine, Faculty of Medicine, University of Tsukuba, Tsukuba, Japan

## Abstract

The combined squamous cell carcinoma (SCC) with neuroendocrine (atypical carcinoid (AC)) tumor is extremely rare in the head and neck. We present here the first case of SCC with AC arising in the floor of the mouth of 65-year-old man. The tumor is comprised of two components of SCC and AC in the biopsy specimen. Neuroendocrine tumor component was classified as AC from the punctate necrosis and 2–10>/10 HPF. Immunohistochemical staining was HMW-CK/34B (+) and P63 (+) in SCC and synaptophysin (+) and CD56 (+) in AC. The pathological diagnosis of SCC with AC was made from both the morphological and immunological exam. Concurrent chemoradiotherapy was performed with radiotherapy 70.2 Gy and chemotherapy of CDDP and VP-16. Although the treatment effect was complete response both of primary tumor and of neck metastases, the recurrence of the primary tumor was after 6 months. Bilateral modified radical neck dissection and tumor resection of the floor of the mouth with reconstructive surgery of anterior lateral thigh free flap were performed. Although the primary and neck tumor did not recur, the multiple lung metastases and mediastinum lymph node metastases occurred at 6 months after surgery.

## 1. Introduction 

Neuroendocrine neoplasms are a heterogeneous group of tumors that vary from benign to highly malignant. WHO (2005) classified neuroendocrine tumor (NET) of the larynx into 4 types: (1) typical carcinoid, (2) atypical carcinoid (AC), (3) small cell carcinoma, neuroendocrine type, and (4) combined small cell carcinoma, neuroendocrine type, with non-small cell carcinoma [1]. The AC (synonyms of malignant carcinoid, moderately differentiated neuroendocrine carcinoma, and large cell neuroendocrine carcinoma) is the most frequent, constituting 54% of all NET in this site, followed by the small cell carcinoma, neuroendocrine type (34%), paraganglioma (9%), and the typical carcinoid (3%) [1]. Although the NET is a tumor that occurs particularly in the lung and larynx, oral cavity is a rare site for a primary NET [2]. Recently, neuroendocrine differentiation has also been found in some tumors not considered to be of neuroendocrine origin, including squamous cell carcinoma (SCC) of the lung and esophagus [3, 4]. The occurrence and possible role of NET in the head and neck SCC have not yet been analyzed. Combined-type SCC and AC instances in the head and neck area were reported only in 3 cases and very rare [5–7]. We report here the fast case of the combined SCC with AC of the floor of the mouth.

## 2. Case Report

**Figure 1:**
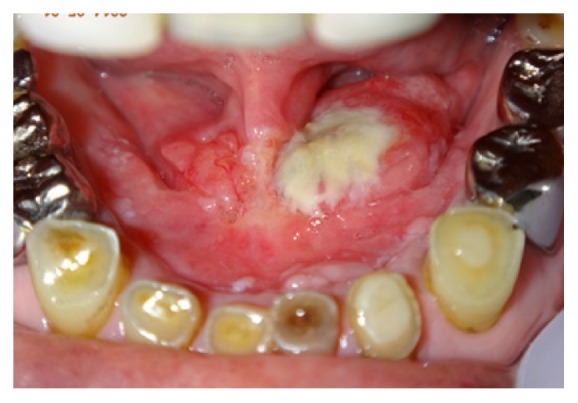
Intraoral examination shows relatively well defined elastic hard mass with ulcer in the left floor of the mouth, which measures approximately 36 × 33 mm.

A 65-year-old Japanese man referred to the Department of Oral and Maxillofacial Surgery, University of Tsukuba Hospital, complaining of pain in the floor of the mouth for one month. His medical history revealed diabetes mellitus, hypertension, chronic pancreatitis, reflux esophagitis, and iron deficiency anemia. His face was symmetrical and there was no trismus. The regional lymph nodes were swollen multiply in both sides from level I to level II. Intraoral examination shows relatively well defined elastic hard mass with necrotic ulcer in the right to left floor of the mouth, which measures approximately 36 × 33 mm (Figure 1).

**Figure 2:**
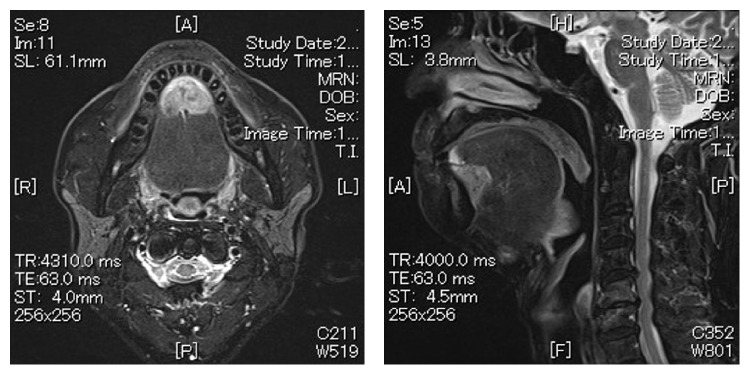
T2 weighted MRI sequence shows a 29 × 23 × 22 mm heterogeneous high signal mass in the floor of mouth.

**Figure 3:**
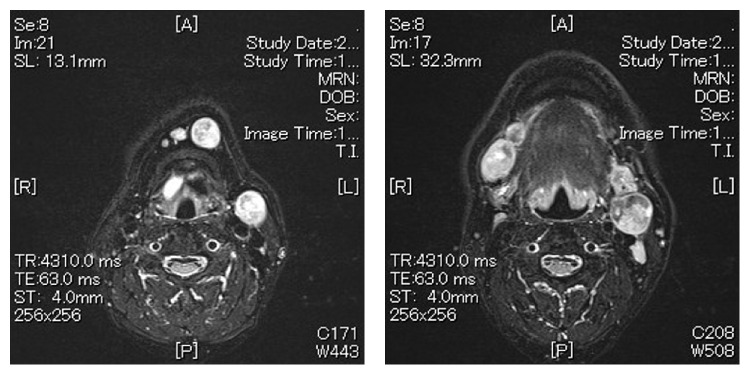
Bilateral multiple neck lymph node metastases are shown. The level Ia LNs are swollen in 16 mm and 7 mm, right level Ib LNs are swollen in 23 mm and 13 mm, left level Ib LN is swollen in 5 mm, and left level IIa LN is swollen in 37 mm.

T2 weighted MRI showed a sequence that shows a 29 × 23 × 22 mm heterogeneous high signal mass in the floor of mouth (Figure 2). Bilateral multiple neck lymph node metastases are depicted in MRI. The level Ia LNs are swollen in 16 mm and 7 mm, right level Ib LNs are swollen in 23 mm and 13 mm, left level Ib LN is swollen in 5 mm, and left level IIa LN is swollen in 37 mm (Figure 3). The 18F-fluorodeoxy-glucose positron-emission tomography combined with computed tomography (18F-FDG PET/CT) revealed FDG uptake in the floor of the mouth mass measuring 28 × 13 mm with the SUV max 10.4 and bilateral multiple LNs.

**Figure 4:**
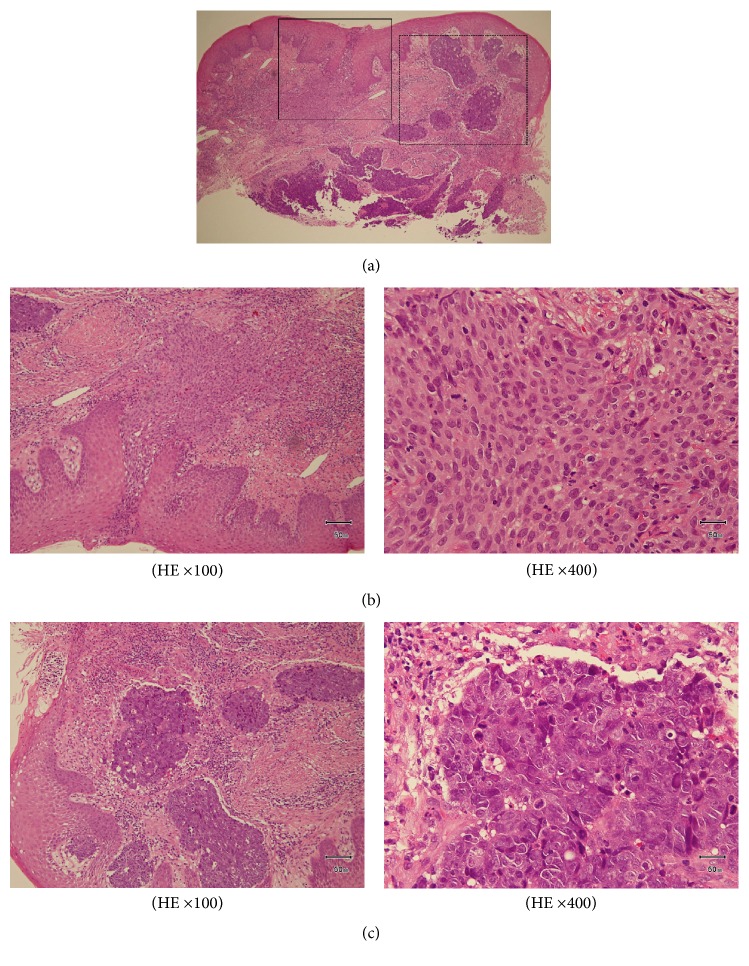
(a) The histopathology of biopsy specimen (HE ×40). The square of straight line was comprised of SCC, and dotted line was comprised of AC. Microscopically, the tumor consists of two components of SCC and AC. (b) The component of SCC. Nonkeratic dysplastic squamous cells proliferated with apoptosis and mitosis. (c) The component of AC. The cells change in a larger way and have high N/C rate and chromatin, a lot of mitosis and karyolysises are seen. A punctate necrosis is observed in the tumor nest, and the rosette structures are observed.

**Figure 5:**
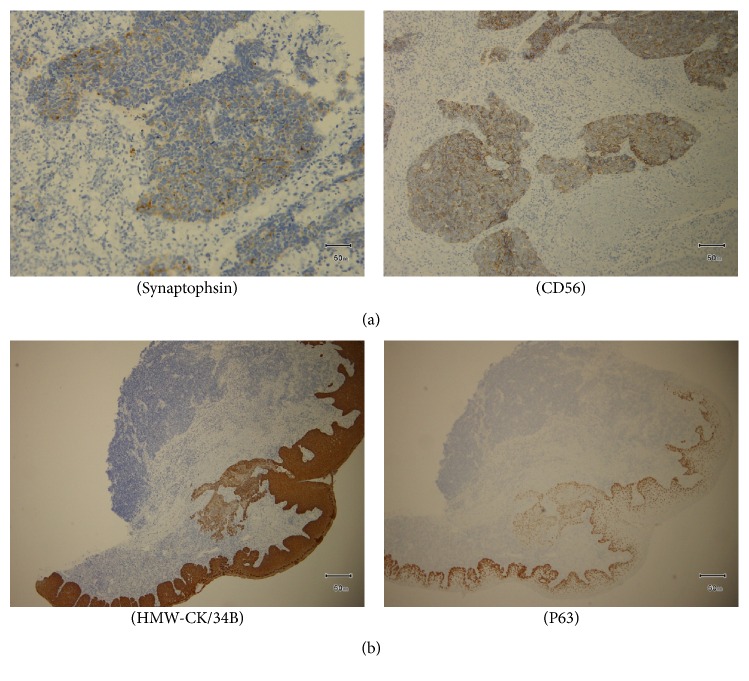
(a) Immunohistochemical staining shows that the tumor cells are positive for synaptophysin and CD56 in SCC. (b) Immunohistochemical staining shows that the tumor cells are positive for HMW-CK/34B and P63 in SCC.

The incisional biopsy was performed from floor of the mouth under local anesthesia. Microscopically, the tumor consisted of two components of SCC and AC. SCC consisted of nonkeratic dysplastic squamous cells proliferated with apoptosis and mitosis. The cells change larger and have high N/C rate and chromatin, a lot of mitosis and karyolysises are seen in component of NET. The punctate necrosis was observed and 2–10>/10 HPF. The NET component was classified as AC (Figures 4(a)–4(c)). Immunohistochemical staining was synaptophysin (+), CD56 (+), and chromogranin A (−) in AC and HMW-CK/34B (+) and P63 (+) in SCC. There was no transitional part between SCC and AC (Figures 5(a) and 5(b)). From these findings pathological diagnosis of SCC with AC in the floor of the mouth was made.

Concurrent chemoradiotherapy was performed with radiotherapy 70.2 Gy and chemotherapy of CDDP and VP-16 for 4 times under the consideration of unresectable neck metastases. Chemotherapy regimen was day 1: CDDP 70 mg/m^2^ + VP-16 100 mg/m^2^, day 2: VP-16 100 mg/m^2^, and day 3: VP-16 100 mg/m^2^. The highest side effects according to CTCAE ver. 4.0 were leukocytopenia (G4), anemia (G3), and thrombocytopenia (G3). The aspiration pneumonia occurred during pancytopenia after second chemotherapy. The leukocyte counts recovered on administrating of G-CSF and aspiration pneumonia was improved with the administration of antibiotics. The treatment effect was complete response both of primary tumor and of neck metastases.

**Figure 6:**
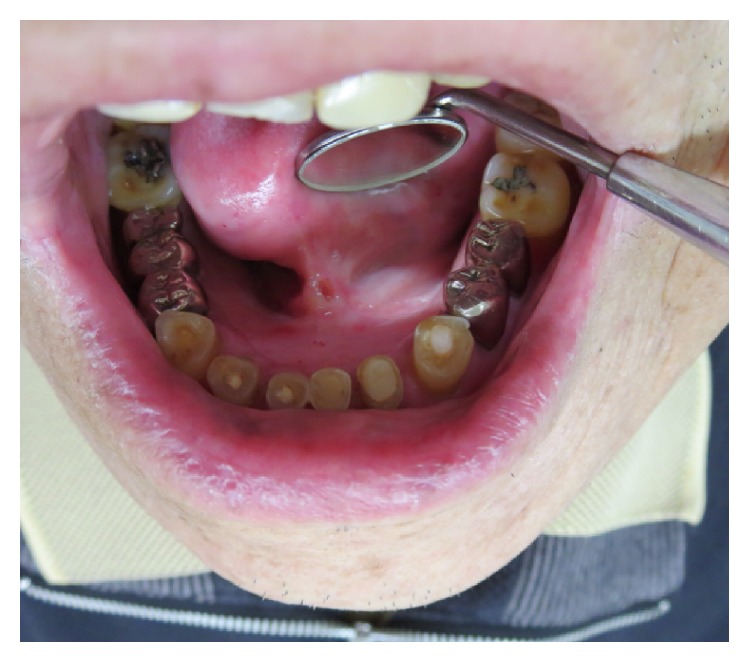
Intraoral examination 6 months after chemoradiotherapy shows small ulcer in the left floor of the mouth.

**Figure 7:**
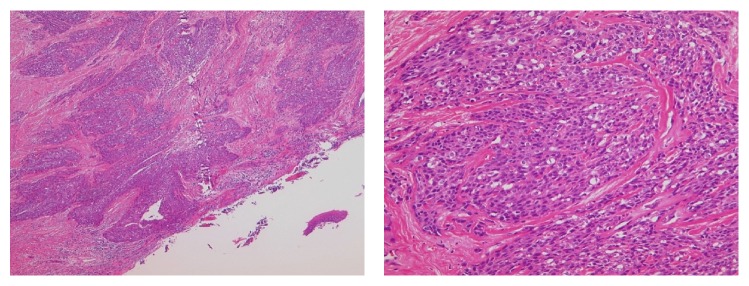
The histopathology of recurred resected specimen (HE ×40, ×100). Microscopically, the resected tumor of floor of mouth was SCC.

**Figure 8:**
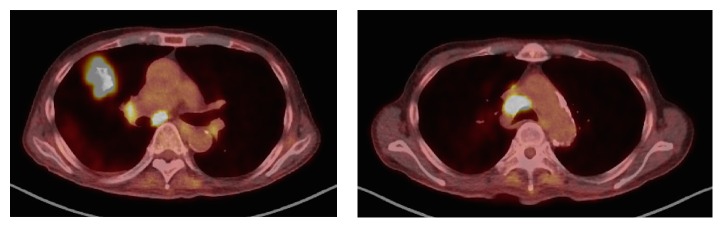
Lung metastasis and mediastinum lymph node metastases are depicted in the PET-CT.

The recurrence of the primary site occurred 6 months from the end of chemoradiotherapy with a diagnosis of primary site biopsy (Figure 6). The volume of lymph node metastases was decreased and changed to resectable. Bilateral modified radical neck dissection and tumor resection of the floor of the mouth with reconstructive surgery of anterior lateral thigh (ALT) free flap were performed under general anesthesia. The pathological diagnosis was SCC without AC in the primary site (Figure 7). There were no metastases in the specimen of neck lymph nodes. Although the primary and neck tumor did not recur, the multiple lung metastases and mediastinum lymph node metastases were diagnosed with FDG PET at 6 months after surgery (Figure 8). The patient received best supportive care with chemotherapy of paclitaxel and cetuximab.

## 3. Discussion

NETs represent a rare, heterogeneous subset in the laryngeal malignancies and are classified into distinct groups and ranging from benign to highly malignant. The oral cavity is a rare site of a primary NET and only 12 cases were reported [2]. Neuroendocrine differentiation has recently been reported in SCC of the lung and esophagus. The occurrence and possible role of neuroendocrine differentiation in the head and neck SCC have not yet been analyzed [8]. It has been hypothesized that tumor cells with neuroendocrine characterization may produce peptides to stimulate tumor growth via autocrine or paracrine mechanisms [4]. Three previously reported SCC with AC of head and neck cases were larynx, maxillary sinus, and upper gingiva [5–7]. The present case was the first report represented on the floor of the mouth with a composite tumor consisting of SCC and AC. Although the composite tumor consisted of combined SCC and small cell carcinoma was reported sometimes, to our knowledge, our case is the fourth to document a composite tumor including AC [5, 7].

A capacity for multidirectional differentiation could arise from pluripotent stem cells. SCC and AC could have arisen from pluripotent cells that differentiated along two distinct paths or the AC could have differentiated secondarily from cells arising in SCC [5, 9]. Another hypothesis is that AC derived from pluripotential indifferent cells of either the squamous epithelium or the minor salivary gland [10]. In the present case, there was no transitional part between SCC and AC in the biopsy specimen, suggested to arise from pluripotent cells that differentiated along two distinct paths.

Immunohistochemically, NET frequently expresses chromogranin A, synaptophysin, and CD56. The tumor cells of NET component are positive for synaptophysin and CD56 in our case. The SCC component was negative for synaptophysin and CD56 and positive for HMW-CK/34B and P63. Nisman et al. reported neuroendocrine differentiation in SCC was associated with poor prognosis [3]. On the other hand, chromogranin A and synaptophysin expression were reported not to associate with advanced disease stage and not to affect patient survival [8]. More cases of this tumor need to accumulate to clarify biological behavior and prognosis.

The AC most occur in supraglottic submucosal in the sixth- and seventh-decade males (M : F, 3 : 1). The rate of metastases was reported 66.7% and the 5-year survival 46% [10]. There was no evidence for the treatment of head and neck AC. The primary treatment is reported to be the surgery and the radiotherapy, with rare response in the chemotherapy [10]. However, AC is reported to be relatively resistant to chemotherapy and radiation therapy [11], and there is no proven optimal therapy for metastatic unresectable AC. Although surgical resection is usually recommended, patients did respond to radiotherapy and chemotherapy, suggesting a combined approach may be indicated in the larynx AC [12]. It was reported that the treatment of primary neoplasms consisting of more than one histological type is tailored to the most histologically aggressive tumor [7]. In our case, the clinical stage was advanced with bilateral multiple neck lymph node metastasis and the chemoradiotherapy was selected because of unresectable and aggressive tumor feature.

Treatment regimens showing efficacy in pulmonary carcinoid were reported to include octreotide-based therapies (10% response rate (RR), 70% disease control rate (DCR)), etoposide (VP-16) + platinum (23% RR, 69% DCR), and temozolomide-based therapies (14% RR, 57% DCR) [13]. The regimen for our case was selected as etoposide + platinum, because platinum is standard regimen for SCC and both effective for SCC and effective for AC. Fortunately the chemotherapy with CDDP and VP-16 and radiotherapy were effective and achieved complete response. The side effect for CDDP and VP-16 of grade 4 leukocytopenia occurred after chemotherapy and the aspiration pneumonia occurred during pancytopenia. This chemotherapy was tolerable because the leukocyte counts recovered on administrating of G-CSF and aspiration pneumonia was improved with antibiotics.

Although the treatment effect was complete response, the recurrence of the primary site occurred 6 months from the end of chemoradiotherapy. The resected primary tumor was SCC without AC, and there were no tumors in the lymph nodes. In the reported combined SCC and AC of the lung, intermediate-grade AC is considered as less aggressive than SCC. The rapid disease progression was suggested that SCC component contributes to the metastasis [14]. In the present case, the component of AC suggested to metastasize to the lymph nodes and to be sensitive to chemoradiotherapy. The component of SCC in primary site was not sensitive for chemoradiotherapy and recurred.

We experienced the first case of SCC with AC of the floor of mouth. More cases of this tumor need to accumulate to clarify biological behavior, treatment, and prognosis. Moreover the occurrence and possible role of SCC with AC have not yet been analyzed, and further research will be desired in the future.
